# Progress in Medicine

**Published:** 1899-01-14

**Authors:** 


### Progress in Medicine.

### RENAL MEDICINE.

Two cases of acute Bright's disease are discussed by W. Carter[1] as illustrating the methods of treatment of uræmia. Both patients were young adults, the attacks followed exposure, and large quantities of albumen were present. The writer is opposed to any hard and fast rule of treatment, holding that each case of uræmia should be managed according to circumstances. Thus, to one patient who was partly comatose he gave oxygen inhalations, and to the other jaborandi and hot drinks with success. Everett J. Brown,[2] in speaking of the extreme difficulty of diagnosing interstitial nephritis, relies chiefly on the heart and general symptoms. If albumen be absent he measures the urine for 24 hours, and takes the sp. gr. and the amount of urea. When there are more than 60 ounces of sp. gr. less than 1,018, and under 450 grains of urea, he adds, we may conclude in favour of that disease. The phosphates are also reduced, and their amount may be estimated with sulphate of magnesium in the centrifuge. As to treatment, he allows no meat or eggs, but fish, and occasionally poultry, plenty of fat, milk and water, moderate amounts of starchy and saccharine food, with warm clothing and regulation of the bowels. The two objects to be aimed at are the removal of the irritating poison from the system, and relaxation of the arterial contraction. Life may be prolonged indefinitely by care.

**Uræmia.**—Bruner[3] finds that the blood in chronic nephritis is remarkably diluted, and that especially is this the case in uræmia. Œdema is a compensatory process, removing the excess of water from the blood, and so preventing the uræmic state. He notes also the deficiency of sodium salts, which, perhaps, we should aim at restoring in our treatment. Insanity is a rare sequela of uræmia, as Bischoff[4] mentions, and may follow either from the intoxication itself or from the convulsions it produces. It usually takes the type of acute mania, together with disturbances akin to the paralytic seizures of general paralysis, from which it may be difficult to distinguish it. Sir Grainger Stewart's recent lecture[5] on uræmia lays stress on the fact that attacks may come on when the kidneys are recovering, and excreting a fair amount of urea and water, if some slight disturbance takes place in the body increasing the amount of waste products from the muscles. The kidneys being unable to respond to the strain on them, poisoning takes place. In a case given by him some abrasions and superficial ulceration of the legs occurred in a patient who was recovering from a kidney attack, slight endocarditis followed, and uræmia resulted in spite of the activity of the kidneys. A drinking bout, septic absorption from intestinal ulcers, an injury or hæmorrhage, may be sufficient to upset the balance and lead to this state. When uræmia appears an extraordinary increase of waste tissue products is found in the blood, although the excretion may be going on at nearly the normal rate. A curious contrast to this increased metabolism is seen in the excretion of the healthy body during great exertion. Bookman[6] examined the urine of a competitor in a six days' cycle race, who had only five hours' sleep during the time. There was a reduction of urea, uric acid, sodium chlorides, phosphates, and water, perhaps from the perspiration, but no other evidence of change. The toxicity of the urine was less than normal, and yet no after effects were found to indicate a retention in the body of a great quantity of waste products.

The interpretation of hæmaturia as a symptom of kidney disease is discussed at great length by D. Newman.[7] The following questions may be asked: (*a*) What is the colour of the blood? (*b*) Is the whole stream uniformly coloured? (*c*) Is the blood increased by exercise? (*d*) Are there clots, and what is their shape? (*e*) Are there tube casts or other deposits present? (*f*) Are the attacks of bleeding of short duration, or continued? The colour, indeed, may be affected by the previous condition and alkalinity of the urine even more than by the source of the bleeding. The clots, too, may be so acted on by the urine as to be mistaken for fragments of a new growth, but even very large clots are not to be regarded as due necessarily to a bladder lesion, for they are sometimes caused by kidney disease, and occasionally casts of the pelvis as well as of the ureters may come away. A hæmorrhage increased by moving about is often due to calculus, pressure of a tumour on the renal veins, or to a movable kidney, while one which persists in spite of complete rest may point to malignant or tubercular ulceration. Much can be learnt from cystoscopy, especially if the blood can be seen coming from one or both ureters. The presence of albumen in excess of that due to the blood present indicates renal disease, and to measure this Newman compares the quantity with the hæmoglobin present. If the proportion is much more than 1 to 1·6 we may infer kidney mischief. The writer has collected many curious instances of hæmorrhages due to pressure on or torsion of the renal veins, or to reflex spasm from catheterisation, from calculus in one of the ureters, from hydronephrosis, or from acute abdominal affections. In renal tubercle there may be a congestive hæmorrhage at early stages as well as that from tissue destruction later on. Newman lays stress on the cloudy viscid urine with renal debris and pus, but without tube casts, as indicative of tubercle. The bacillus should, however, be searched for, and the cystoscope employed to show whether one or both kidneys are affected. In cystic degeneration, he finds hæmaturia during the latter stages in a fourth of his cases, sometimes scanty and in other attacks profuse. Casts, excess of albumen, uræmia, and heart changes may also be found here. In malignant disease, bleeding is late in commencing, but is afterwards more profuse, continuous, and progressive than that from other causes. The presence of detached "cancer cells" is of no diagnostic value, but occasionally fragments of a growth may be found in the urine.

C. A. Herter[8] discusses the methylene blue test for delay of the kidney in excreting effeta products, and comes to the following conclusions: (*a*) A distinct delay in the excretion of the blue shows inability of the kidney to do its work in due time; (*b*) when this is very marked it is evidence of commencing uræmia;

(c) periods of delay may alternate with normal excretion; (d) rapid disappearance of the colour shows that the kidney excretes normally even though albumen and casts be present. He regards many forms of acute nephritis as due to bacillary infection. Tomlinson[9] refers to the usefulness of the test, and mentions the value of the drug as a therapeutic agent in the early stages of parenchymatous nephritis, and of renal inadequacy.

[1] Liverpool Med. Chir. Jour., July. [2] Med. Rec., Sept. 3. [3] Brit. Med. Jour., Sept. 17. [4] Brit. Med. Jour., Aug. 6. [5] Practitioner, Aug. [6] Med. Rec., Aug. 6. [7] Lancet, July 2, 9, 16. [8] Med. Rec., Nov. 5. [9] Amer. Med. and Surg. Bull., May 25.

